# Negative [99mTc]Tc ‐DPD Scintigraphy, Presence of Monoclonal Protein and Biopsy Suggestive of AL Amyloidosis in a Patient With Homozygous p.Ala101Val Transthyretin Gene Variant

**DOI:** 10.1002/ccr3.71928

**Published:** 2026-02-24

**Authors:** Paulina Kryszpin, Piotr Jachimowski, Łukasz Augustowski, Mateusz Ziarkiewicz, Grzegorz Basak, Marta Lipowska, Marta Legatowicz‐Koprowska, Bogna Ziarkiewicz‐Wróblewska, Monika Gawor‐Prokopczyk

**Affiliations:** ^1^ Medical University of Warsaw Warsaw Poland; ^2^ Department of Hematology, Transplantology and Internal Diseases Medical University of Warsaw Warsaw Poland; ^3^ Department of Neurology Medical University of Warsaw Warsaw Poland; ^4^ Department of Pathomorphology, National Geriatrics Rheumatology and Rehabilitation Institute Warsaw Poland; ^5^ Department of Pathomorphology Medical Center of Warsaw Medical University Warsaw Poland; ^6^ Department of Cardiomyopathy National Institute of Cardiology Warsaw Poland

**Keywords:** AL amyloidosis, Ala101Val (c.302C>T) variant, ATTR amyloidosis, ATTRv, cardiac amyloidosis

## Abstract

Amyloidosis is a rare disease associated with the deposition of misfolded proteins that damage multiple organs, leading to a wide range of symptoms. The most frequently implicated proteins in amyloidosis include immunoglobulin Free Light Chains (FLC), related to AL amyloidosis, and transthyretin (TTR), which is responsible for ATTR amyloidosis. Here, we report the case of a 52‐year‐old patient with a history of chronic diarrhea, loss of weight, and orthostatic hypotension with imaging‐confirmed cardiac amyloidosis (CA). The diagnostic process was notably complex due to elevated serum FLC, negative [99mTc] DPD scintigraphy, and inconclusive immunohistochemical typing of amyloid fibrils in oral mucosa. The ambiguity of the case prompted genetic analysis, which revealed a rare homozygous p.Ala101Val (c.302C>T) variant in the TTR gene, leading to the diagnosis of hereditary ATTRv amyloidosis. During follow‐up, sensorimotor neuropathic symptoms developed in addition to the pre‐existing autonomic neuropathy; consequently, Tafamidis therapy was initiated, leading to stabilization of the disease. This case report highlights the diagnostic challenges in distinguishing AL from ATTR amyloidosis, simultaneously pointing out the limitations of current noninvasive testing methods. The importance of genetic testing was demonstrated by the identification of a previously reported pathogenic variant. Furthermore, this represents the first documented case of a homozygous variant associated with a prominent cardiological and neurological phenotype. It is crucial to consider amyloidosis in the context of a pattern of cardiovascular and neuropathic manifestations, as well as to employ appropriate diagnostic approaches to establish an accurate diagnosis and guide optimal management.

## Key Clinical Message

- Biopsy‐confirmed amyloidosis with coexisting hypertrophic cardiomyopathy, negative [99 mTc]Tc ‐DPD scintigraphy, and a detectable monoclonal protein is highly suggestive of AL amyloidosis. Nevertheless, certain ATTRv variants, such as the homozygous p.Ala101Val, may present with an identical profile. In equivocal cases, genetic testing is essential to avoid diagnostic delays.

## 1 Introduction

Transthyretin amyloidosis variant (ATTRv) is an extremely rare genetic condition caused by mutation in the TTR gene and aggregation of transthyretin, leading to the formation of amyloid fibrils [1]. Amyloid is deposited in various organs, resulting in their damage and a wide range of symptoms. Over 140 pathogenic variants of the TTR gene connected with ATTRv have been reported [2]. What is important is that most of the described pathogenic variants are associated with a predisposition for a specific clinical presentation as a cardiomyopathy, neuropathy, or mixed phenotype [3].

Broad spectrum of symptoms, rarity of the disease and frequent lack of access to appropriate diagnostic methods lead to irreversible changes and delayed diagnosis, resulting in worse prognosis [4]. Clinical manifestations of cardiac involvement are not distinctive, but on the other hand correlation of additional symptoms and family history of the disease may facilitate early diagnosis. The diagnostic process is based on non‐fully sensitive and specific radiological examinations (cardiac scintigraphy and MRI), which should be supplemented in equivocal cases by tissue biopsy with potential complications [5].

Another, more common cause of amyloidosis is AL (light chain) amyloidosis in which amyloid fibrils are composed of immunoglobulin light chains produced by monoclonal plasma cell clones [1]. It is important to distinguish between these two types of amyloidosis due to differences in treatment and prognosis [6]. Undoubtedly, genetic testing facilitates precise diagnosis in ambiguous cases and enables earlier detection, which in turn leads to a better prognosis. Here, we present the diagnostic dilemmas in cardiac amyloidosis (CA), exemplified on an extremely rare case of ATTR with p.Ala101Val (c.302C>T) mutation.

## 2 Case History

Fifty two years old female patient presented to the hospital with a history of chronic diarrhea, 20 kg weight loss and orthostatic hypotension with periodic blood pressure drops to 70/55 mmHg combined with syncope. Previous gastroenterological diagnostic procedures had not revealed any organic cause for the diarrhea; therefore, irritable bowel syndrome had been identified. During the diagnostic process, features of hypertrophic cardiomyopathy were described in echocardiography, subsequently confirmed by cardiac magnetic resonance imaging (cMRI). After a year of persistent symptoms, a suspicion of systemic amyloidosis was raised. Blood tests revealed elevated serum Free Light Chains (sFLC) but without presence of monoclonal protein, elevated NT‐proBNP and troponin T concentration and mild normocytic anemia (see Table 1). Additionally, increased amounts of protein were detected in the urine, but no monoclonal protein was noticed. The cardiac scintigraphy with radioactive technetium‐99 m (^99m^Tc) and 3,3‐diphosphono‐1,2‐propanodicarboxylic acid (DPD) did not reveal increased cardiac uptake (stage 1 in Perugini score) [7]. The general clinical presentation strongly implied AL amyloidosis. However, the histopathological examination and flow cytometry of the bone marrow trephine biopsy revealed polyclonal plasma cells (CD138+, CD38+, κ+, λ+, CD56‐) and absence of amyloid deposition.

**TABLE 1:** Laboratory parameters.

Parameter	First hospitalization	Second hospitalization	Normal value
HGB (g/dl)	11.30	8.00	12.00–16.00
RBC (mln/μL)	3.65	2.70	3.93–5.22
MCV (fL)	96.00	95.00	80–97
WBC (tys/μL)	4.33	4.90	3.98–10.04
NEUT (tys/μL)	1.81	1.98	2.00–7.00
LIMF (tys/μL)	2.05	2.52	1.00–3.00
PLT (tys/μL)	261	229.00	150–400
sFLCκ (mg/L)	39.34	67.58	3.3–19.4
sFLCλ (mg/L)	32.55	43.05	5.7–26.3
sFLCκ/FLCλ ratio	1.21	1.57	0.37–3.10
NT‐pro‐BNP (pg/mL)	706.00	1250.00	0.00–125.00
Troponiny T (ng/L)	42.2	96.52	0.00–14.00
eGFR (ml/min/1,73m^2^)	37.2	41.1	> 90.00
Uric acid (mg/dL)	6.6	3.80	2.4–5.7
Total proteins (g/dL)	5.95	6.50	6.4–8.3
ALB (g/dL)	3.43	3.70	4.0–4.8
α 1‐GLB (g/dL)	0.26	0.30	0.2–0.4
α 2‐GLB (g/dL)	0.64	0.60	0.5–0.9
β‐GLB (g/dL)	0.43	0.31	0.34–0.52
Γ‐GLB (g/dL)	0.89	1.30	0.8–1.4
Urine protein	97.37	9.00	0–15

## 3 Differential Diagnosis, Investigations and Treatment

**FIGURE 1:**
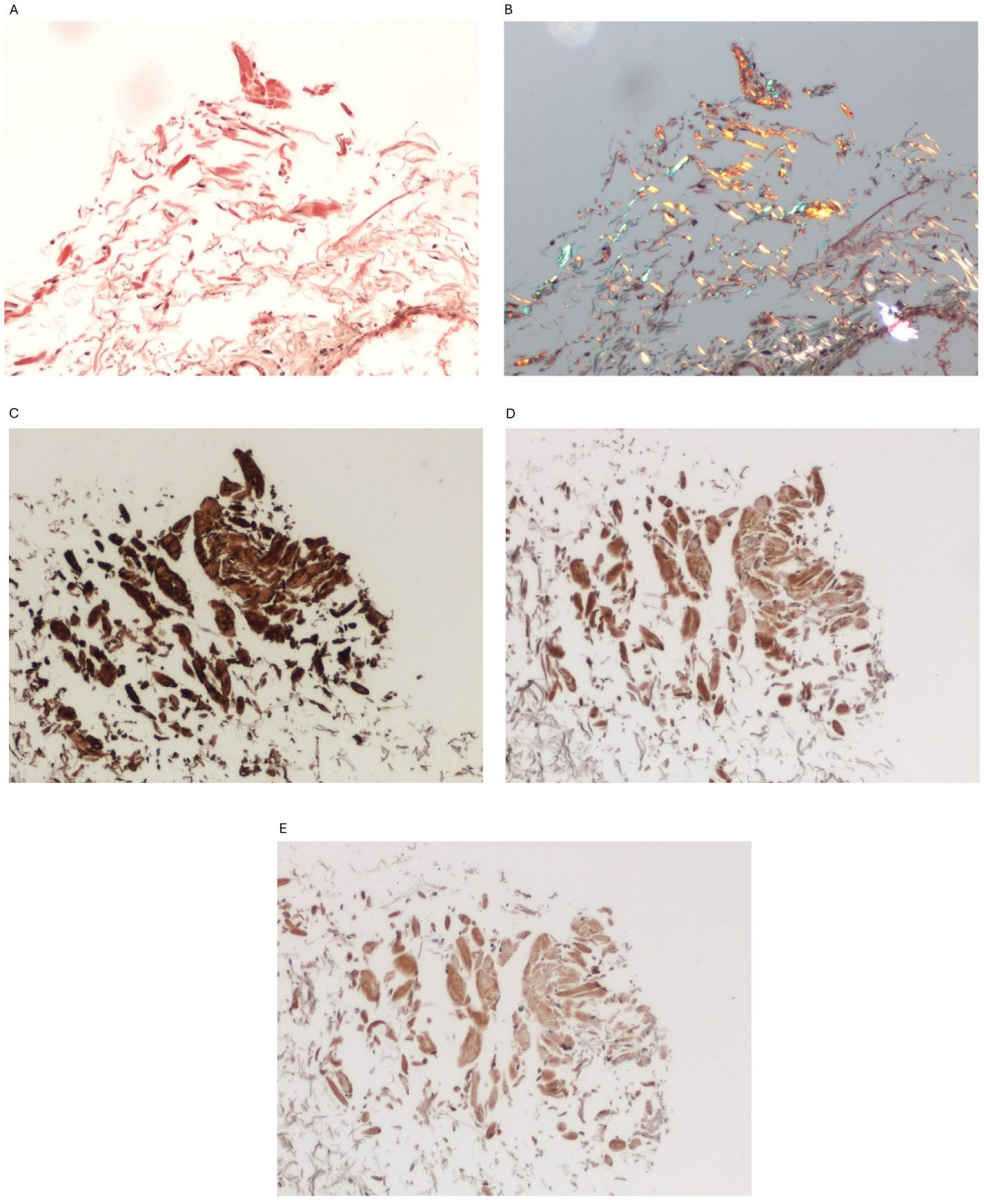
Oral mucosa. (A) Congo red – deposits of amyloid stain red. (B) Congo red – apple green birefringence on polarization. (C) Immunohistochemistry anty‐TTR: Strong reaction – dark brown. (D) Immunohistochemistry anti‐lambda: Positive reaction – brown. (E) Immunohistochemistry anti‐kappa: Slight reaction – light brown.

**FIGURE 2:**
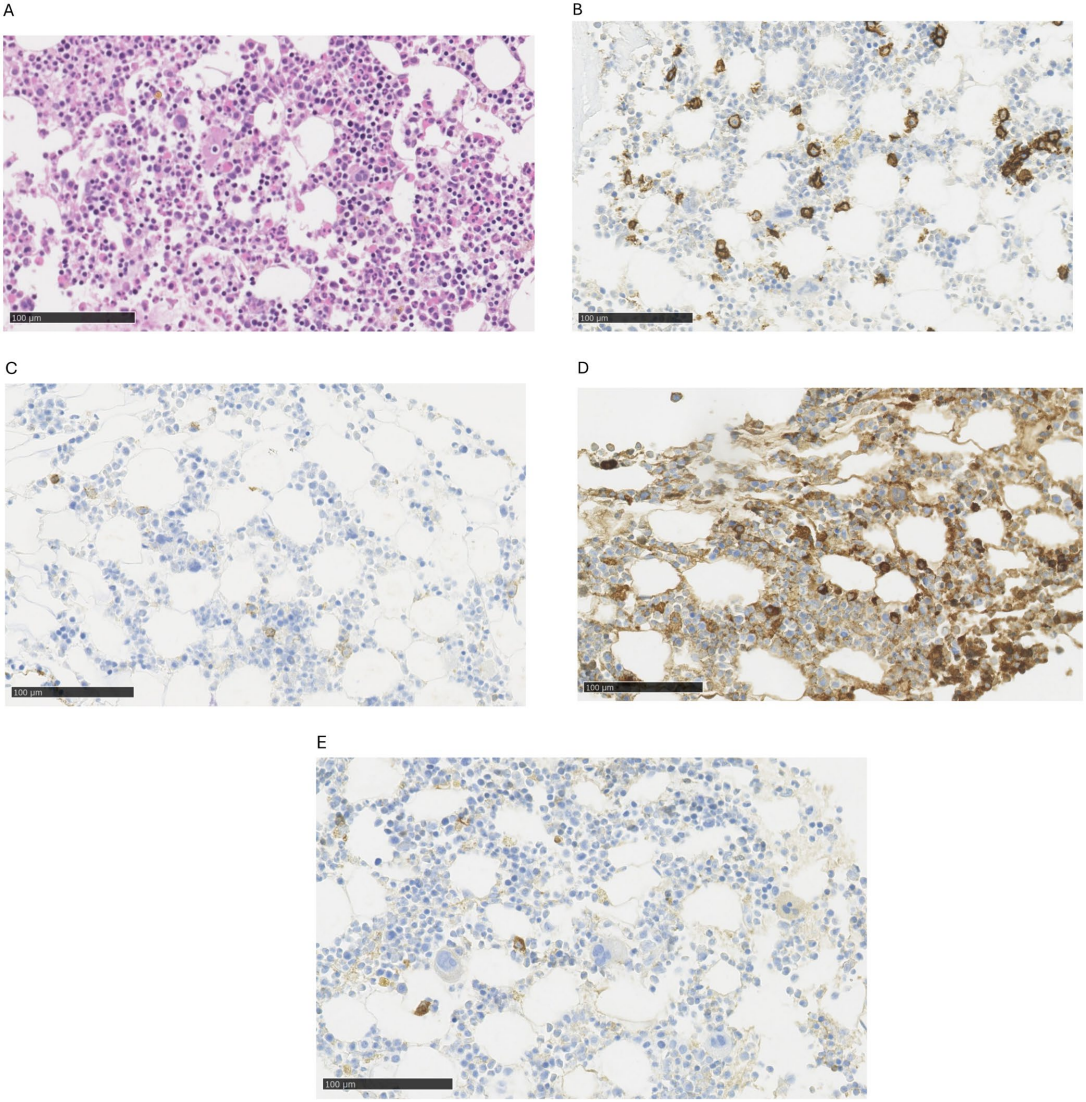
Bone marrow biopsy. (A) Hematoxylin and eosin stain. (B) Immunohistochemistry: CD138 stain scattered plasma cells and one perivascular cluster. (C) Immunohistochemistry: Some weakly CD56‐positive plasma cells. (D) Immunohistochemistry: Kappa‐positive plasma cells. (E) Immunohistochemistry: Single lambda‐positive plasma cells.

Due to persistent symptoms, further diagnostics were carried out at a reference cardiology and hematology center. A repeated cMRI with gadolinum showed intramuscular disseminated late gadolinum enhancement (LGE) suggestive of CA. Nevertheless repeated scintigraphy did not show marker deposition in cardiac location (Perugini 0) [7]. The patient's laboratory tests displayed more severe anemia, increased sFLC, NT‐proBNP and troponin T. (see Table 1) Additionally trace amounts of serum monoclonal IgGλ was detected. Histopathological examination of oral mucosa revealed Congo red‐stained small amyloid nodules with apple green birefringence on polarization light within the interstitial tissue (Figure 1A,B), while immunohistochemical (IHC) typing showed a positive reaction with transthyretin, λ light chains, and a slight reaction with κ light chains (Figure 1C–E). Fine needle biopsy of fatty tissue did not reveal any amyloid deposition. Inconsistencies in test results prompted us to repeat bone marrow biopsy, which showed an increased percentage of polyclonal plasma cells (10%) with positive staining for both κ and λ light chains (with predominance of κ‐positive cells) (Figure 2A–E). Additionally, perivascular deposits fluorescing green in polarized light were found, corresponding to amyloid deposits. Nevertheless, histopathological examination of colonic and gastric mucosal layers did not exhibit amyloid deposition.

**FIGURE 3:**
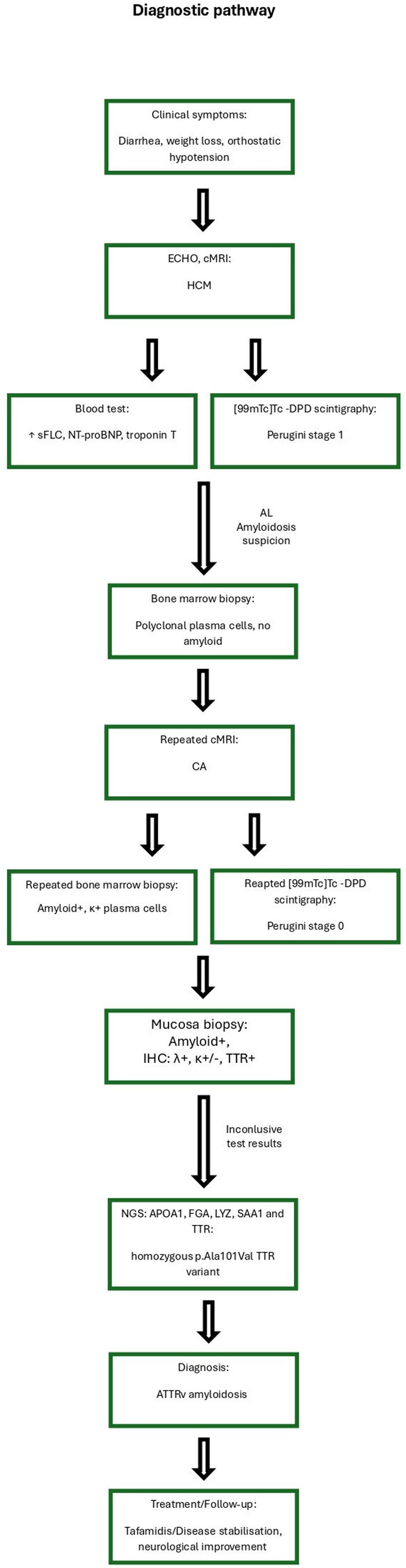
Diagnostic pathway.

Because of unreliable immunohistochemical differentiation of amyloid fibers, as well as discrepant results regarding the existence of plasma cell clone, Next Generation Sequencing (NGS) was performed, including the following genes: APOA1, FGA, LYZ, SAA1, and TTR, which revealed the homozygous p.Ala101Val (c.302C>T) TTR variant (see Figure 3).

The patient was admitted to the neurology department after 4 years from the beginning of symptoms at age 56 to assess neurological involvement and establish further management. Neurological examination revealed tetraparesis more pronounced distally and in the lower extremities, with impaired sensation of pain, temperature, and light touch with a short gloves and stockings pattern. The patient was arising from a chair using arms, was able to walk a short distance independently; for longer distances and outside, she used a rollator or wheelchair.

Electroneurography and electromyography confirmed motor and sensory mixed axonal and demyelinating polyneuropathy with predominance of distal nerve damage, with lack of distal motor responses in peroneal nerves and sensory responses in sural, medial, and ulnar nerves. Electromyography showed neurogenic pattern in distal and proximal muscles of lower limbs and distal muscles in upper limbs. Quantitative Sensory Test confirmed diminished pain and temperature sensation in distal parts of upper and lower limbs.

The clinical picture was dominated by thin fiber neuropathy with autonomic symptoms (mainly diarrhea, orthostatic hypotension, lost bladder control) seen from the beginning of disease. Scales to assess autonomic symptoms were performed and showed abnormalities: in Compass −31 scale 30 points (normal–0 points, range 0 – 100), CADT scale 7 (maximal—normal 16 points, range 0 – 16). Autonomic involvement was followed by thin fiber sensory symptoms‐temperature and pain abnormalities and motor fiber involvement partially masked by heart failure symptoms secondary to HCM. We considered shortage of walking distance in this patient due to mixed cardiologic and neuropathic reasons.

## 4 Conclusion and Follow‐Up

On the basis of these results, the mutated transthyretin amyloidosis (ATTRv) with cardiac and peripheral nervous system involvement was diagnosed. Due to the progression of heart failure and polyneuropathy symptoms, Tafamidis treatment was initiated. Disease stabilization was achieved, along with neurological improvement, manifested by reduced paresis and better autonomic function as indicated by a decrease to 22 points in Compass‐31 scale and increase to 11 points on the CADT scale (Compass‐31 scale 22 points, CADT scale 11 points). The patient's general condition has reached a steady state, as a result of symptomatic cardiac management and effective disease control. The patient will remain under cardiological and neurological follow‐up for ongoing evaluation of the response of Tafamidis treatment.

## 5 Discussion

Amyloidosis is a rare condition which could affect every organ. Without a doubt the most common systemic amyloidosis is AL amyloidosis with an incidence of 12 cases per million people per year [8] In contrast, the much rarer ATTR amyloidosis is estimated to occur at a rate of 0.3 cases per million people per year [6] Concurrence of cardiomyopathy and autonomic dysfunction should always lead to confirmation or exclusion of amyloidosis. Identification of amyloid deposits composed of immunoglobulin light chains, correlated with monoclonal gammopathy, strongly suggests AL amyloidosis [9].

Case report of our patient was previously included in a case series where authors well defined negative scintigraphy and cardiological profile [10] However, the diagnostic difficulties and the inconclusive nature of additional test results prompted us to provide a more comprehensive description of this noteworthy case. We focus specifically on the hematological and pathological diagnostic process and the potential pitfalls that may arise in the form of false test results. Additionally, based on the available literature, we propose possible causes of these phenomena. Furthermore, we describe the neurological profile as well as the patient's evolution after diagnosis and treatment initiation.

In our case we reported elevated levels of sFLC, monoclonal protein in serum which could indicate a plasma cell dyscrasia simultaneously suggest AL amyloidosis as the underlying cause of the disease. Nevertheless, the monoclonal protein was not constantly detected; additionally, both FLC were elevated, while FLCκ/FLCλ ratio remained within the normal range. Interestingly, the prevalence of MGUS (monoclonal gammopathy of undetermined significance) in patients with ATTR amyloidosis is higher compared to the general population [11, 12]. It implies the need for further investigation of equivocal cases. The literature review showed that the occurrence of one type of amyloidosis does not exclude another one [13, 14, 15]. On the other hand, only one published case presented ATTRv with AL amyloidosis [14] suggesting a greater predisposition for the co‐occurrence of AL amyloidosis with wild‐type ATTR, as a much more common condition.

Additionally, bone marrow biopsy revealed predominance of κ‐positive plasma cells correlated with higher levels of sFLCκ. Moreover, a positive IHC reaction with λ light chain in amyloid deposits in the oral mucosa also tangled a diagnosis of AL amyloidosis. In our case, an intraoral salivary gland was chosen due to low invasiveness of biopsy and high sensitivity to detect AL and ATTR amyloidosis [16, 17] However, the result was inconclusive with positive staining for both light chains as well as transthyretin in deposits. In IHC typing of amyloid, it is not uncommon to observe additional accumulation of transthyretin in amyloid deposits originally derived from light chains. Nevertheless, in this case, the IHC reaction was strongest with transthyretin and coexisting light chain positivity could depend on their elevated serum levels (Figure 1C–E) [11] Problems with false positive staining have been widely described [18], indicating the need to perform mass spectrometry (MS) to increase the specificity of the diagnostic process [19].

Imaging tests such as echocardiography and cMRI with LGE may strongly indicate CA. The determination of the type of CA can be achieved without a biopsy in cases of ATTR without MGUS [20]. Invaluable test for the diagnosis of ATTR CA is 99 mTc‐DPD scintigraphy with the sensitivity of positive scan (Perugini cardiac uptake score of 2 or 3) alone of about 95% with 97% specificity [5] Interestingly, in our patient 99 mTc‐DPD cardiac uptake was lower than bone level (Perugini score 1) or absent on another occasion (Perugini score 0) with presence of monoclonal protein, which strongly excludes ATTR amyloidosis. There are published data on correlation between specific TTR variants and reduced sensitivity of 99mTc‐DPD scintigraphy [10, 21, 22, 23, 24, 25, 26]. The authors suggest potential causes of this condition: the early manifestation of the disease without fully developed imaging findings [24, 25]; specific type B of amyloid fibrils composed solely of full‐length TTR molecules [23, 26]. Another potential causes may include the specific binding affinities of radiotracers to amyloid fibrils [24]. In the case of p.Ala101Val TTR variant, lower sensitivity in the homozygous mutations was correlated with both a more severe phenotype and lower scintigraphy sensitivity compared to heterozygous state [10, 21, 22]. Underlying cause remains unknown and indicating the need of further research.

Owing to unavailability of MS at our site, as well as inconclusive hematologic results and suspicion of false‐negative 99mTc‐DPD scintigraphy result we decided to perform sequencing of the most frequently mutated genes associated with hereditary form of amyloidosis [27]. The p.Ala101Val mutation is extremely rare. Only a few cases were described. Moreover, the presented patient is in homozygous state, although ATTRv is autosomal‐dominant disorder, so heterozygous state is sufficient for symptoms to reveal. Lithuanian authors described this mutation in one individual in a homozygous state and in three individuals in a heterozygous state [18]. The age of onset of symptoms in this report ranged from 44 to 74 years. The earliest onset of symptoms was in the individual with the homozygous variant. A history of carpal tunnel syndrome was identified in two individuals. Patients with mutation in heterozygous state presented mainly with cardiomyopathy phenotype, while the individual with the homozygous variant had the most pronounced polyneuropathy with tetraparesis. The authors concluded that homozygous state of this variant was associated with earlier disease onset and neurological involvement compared to the heterozygote state. This observation is consistent with our case with relatively early onset in middle‐age of 52 years and mixed neuropathic and cardiologic phenotype. The female sex is also worth noting, as we observe the predominance of males in polish population [10, 28]. Parents of that patient were relatives, family history revealed no evident ATTRv history. Clinical picture of neuropathy with dominant autonomic symptoms (first symptoms and later marked severity) of thin fiber neuropathy is typical for ATTRv amyloidosis [29]. All symptoms: autonomic, somatic neuropathy and cardiomyopathy were responsible for disability in this patient.

## Author Contributions

**Paulina Kryszpin:** conceptualization, writing – original draft, writing – review and editing. **Piotr Jachimowski:** writing – original draft, writing – review and editing. **Łukasz Augustowski:** conceptualization, writing – review and editing. **Mateusz Ziarkiewicz:** conceptualization, funding acquisition, investigation, supervision, writing – review and editing. **Grzegorz Basak:** funding acquisition, resources. **Marta Lipowska:** investigation, supervision, writing – review and editing. **Marta Legatowicz‐Koprowska:** resources, visualization, writing – review and editing. **Bogna Ziarkiewicz‐Wróblewska:** resources, visualization, writing – review and editing. **Monika Gawor‐Prokopczyk:** investigation, writing – review and editing.

## Funding

This research did not receive any specific grant from funding agencies in the public, commercial, or not for profit sectors.

## Consent

Written informed consent was obtained from the patient to publish this report in accordance with the journal's patient consent policy.

## Conflicts of Interest

The authors declare no conflicts of interest.

## Data Availability

The data that support the findings of this study are available from the corresponding author upon reasonable request.
